# Foreign Body Aspiration in a Child With a Rare Tracheal Bronchus

**DOI:** 10.7759/cureus.26710

**Published:** 2022-07-10

**Authors:** Marzouqi A Salamah, Adel Banjar, Murad Banjar, Abdulrahman Shareefi

**Affiliations:** 1 Medicine, King Abdullah Ear Specialist Center (KAESC) King Saud University Medical City (KSUMC) King Saud University, Riyadh, SAU; 2 Otolaryngology - Head and Neck Surgery, Ohud Hospital, Al-Madinah Al-Munawarah, SAU; 3 Medicine, Rayan Medical College, Al-Madinah Al-Munawarah, SAU

**Keywords:** trachea, bronchoscopy, airway management, foreign body aspiration, tracheal bronchus

## Abstract

A tracheal bronchus is an unusual bronchial division anomaly in which an accessory bronchus arises from the trachea or main bronchus and travels to the higher lobe territory. This report discusses a case of incidentally diagnosed tracheal bronchus after foreign body removal via bronchoscopy.

A one-year-old boy presented to the hospital with cough and noisy breathing after choking on peanuts. On examination, he had mild tachypnea with non-prominent subcostal retractions and diminished airflow in the left lung. Rigid bronchoscopy revealed a foreign body in the trachea at the level of the left main bronchus, which was completely removed in one piece under vision using fiberoptic forceps.

A tracheal bronchus is an unusual congenital abnormality, with most cases being asymptomatic. Appropriate reporting of such anomalies may help healthcare practitioners promptly diagnose, manage, and avoid complications in the tracheal bronchus.

## Introduction

A tracheal bronchus is an unusual bronchial division anomaly in which an accessory bronchus arises from the trachea or main bronchus and travels to a higher lobe territory. Normal tracheobronchial development in humans begins as a median bulge of the anterior pharyngeal wall, which grows at the caudal end of the laryngotracheal groove between 24 and 26 days of gestation. The lung buds usually extended into principal bronchi between days 28 and 30. By day 36, all segmental bronchi are fully structured [1]. Tracheal bronchus has an incidence rate of 0.001% to 2% and was originally reported by Sandifort in 1785 as an unusual anomaly in which an accessory bronchial branch originates higher than the tracheal bifurcation [2]. According to bronchoscopic, autopsy, and radiological studies, the prevalence of right and left tracheal bronchi ranges from 0.1% to 2% and 0.3% to 1%, respectively [3]. Two types of tracheal bronchi have been described; the first is the displaced type, which is associated with the lack of a segmental branch in the superior lobe, and it constitutes a normal segmental bronchial branch of the superior lobe with an unusual origin. The second type is the supernumerary type, which is a genuine accessory bronchus associated with normal upper lobe bronchus branching [2].

The tracheal bronchus is usually asymptomatic; however, it carries the risk of recurrent pneumonia, bronchiectasis, cough, stridor, wheezing, hypoxia, or atelectasis of the altered lobe [4]. Failure or delayed recognition of this anomaly may increase the risk of complications. Herein, we present a case of tracheal bronchus in a one-year-old child incidentally noted during bronchoscopy for foreign body removal. The description of the lesion, clinical features, effect on health, and management are also discussed.

## Case presentation

**Figure 1:**
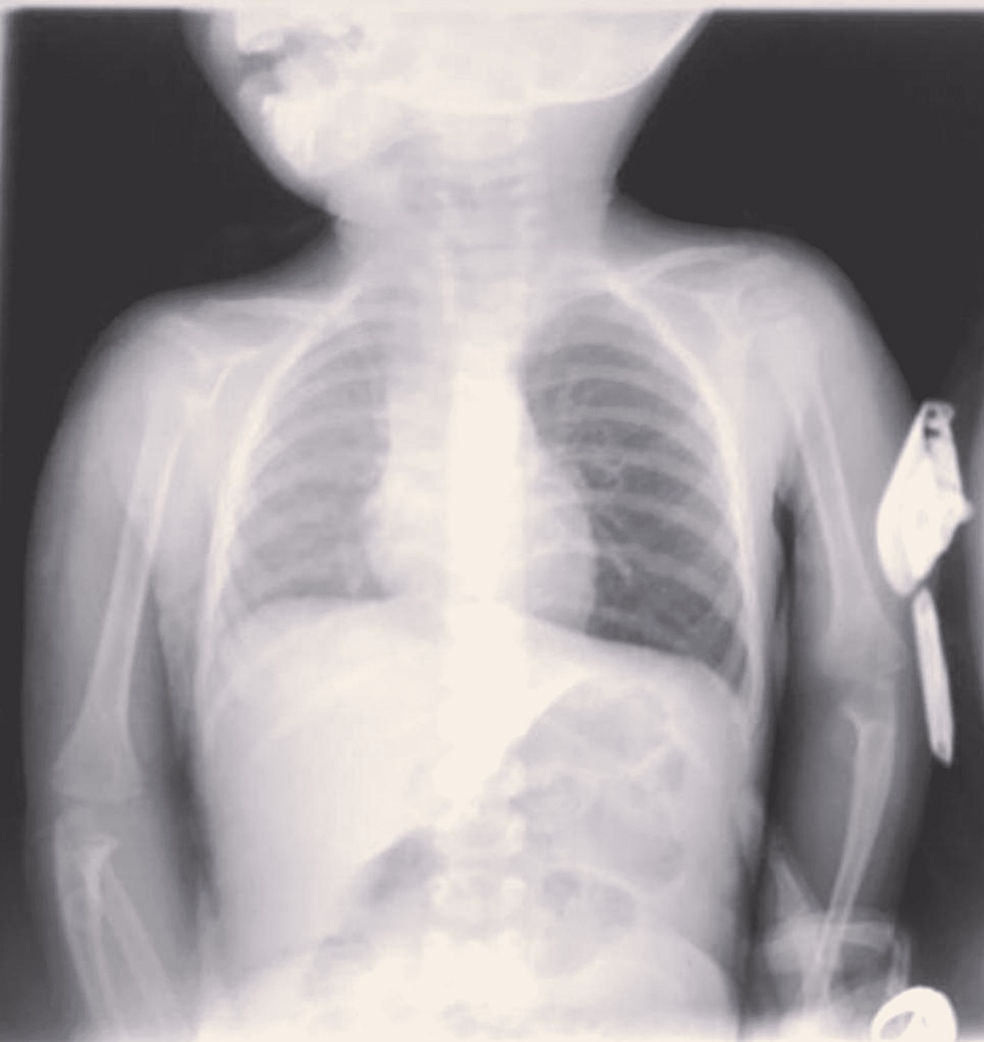
Pre-operative plain chest x-ray showed left lung hyperinflation.

A one-year-old boy with no known medical illnesses was referred to our hospital with a history of cough and noisy breathing within an hour of choking on peanuts. On examination, the child was slightly tachypneic. On inspection of the patient’s chest, mild subcostal retractions were noted. Chest auscultation revealed coarse upper airway sounds and diminished air entry into the left lung. No other abnormal physical examination results were obtained. Chest radiography revealed hyperinflation in the left lung and a diminished right lung volume (Figure 1).

**Figure 2:**
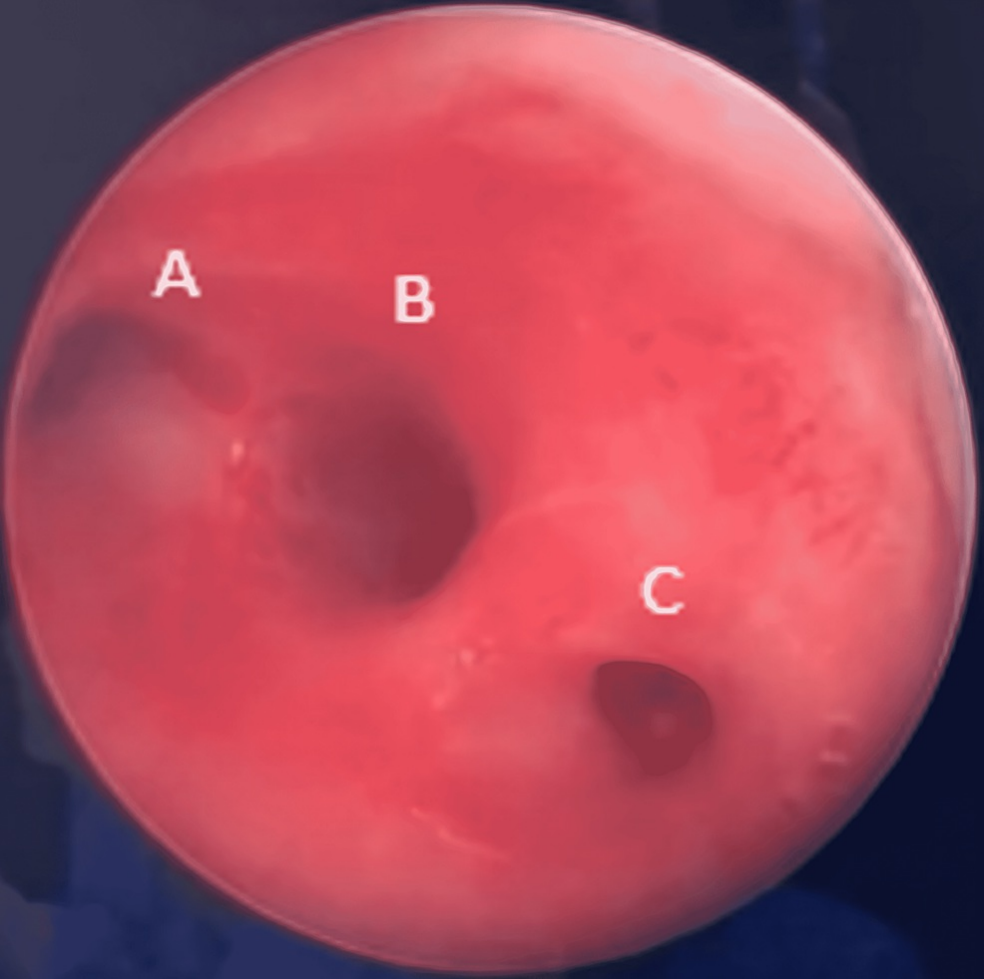
Intraoperative photography after removal of foreign body, re-examination revealed a clear airway with confirmed right tracheal bronchus (A - Left main bronchial orifice, B - Right main bronchial orifice, C - Right tracheal bronchus).

Rigid bronchoscopy revealed a foreign body located in the trachea at the level of the left main bronchus. Moreover, a right accessory tracheal bronchus was observed above the tracheal carina. The foreign body was completely removed under vision using fiberoptic forceps. Further examination of the patient’s airways showed a clear passage with no foreign body and confirmed the lack of a right accessory tracheal bronchus (Figure 2).

The surgery was performed with no postoperative complications, and the patient did not require intubation or oxygen after the procedure. The patient was then transferred to the pediatric intensive care unit for observation. The pediatrician was informed of the anomaly in case the patient needed intubation. The patient was discharged home in a stable and good condition.

## Discussion

A tracheal bronchus is a rare asymptomatic condition that may be associated with difficulties in diagnosis and management. The present report describes a case of tracheal bronchus discovered during bronchoscopy for foreign body removal.

The prevalence of tracheal bronchi among children in the Arabian Gulf was reported in 2021 by Al-Naimi et al. [4]. Of the 1,786 patients who underwent flexible bronchoscopy, only 20 (1.12%) had a tracheal bronchus. The patients’ ages at the time of diagnosis ranged from 2 to 154 months, with a median of 31 months. Most patients with tracheal bronchi (n= 16, 80%) had other associated congenital anomalies; cardiac defects were the most frequently correlated anomalies (8/20, 40%) [4].

While the tracheal bronchus is usually asymptomatic, patients may experience frequent chest infections, retained secretions, stridor, or even foreign body aspiration. Moreover, the inability to identify the tracheal bronchus during intubation can lead to complications. The side of the endotracheal tube may block the tracheal bronchus, leading to lobe atelectasis. In addition, the tracheal bronchus may get intubated unexpectedly, causing pneumothorax or diminished aeration in the remaining part of the bronchopulmonary tree. Therefore, in individuals who exhibit unexplained intraoperative hypoxemia, tracheal bronchus should be considered in the differential diagnosis [5,6].

Because they are usually asymptomatic, tracheal bronchi are usually discovered incidentally on radiographic imaging [7]. An accessory bronchus that originates from the supracarinal area, most probably on the right side, may be noted on a chest radiograph. Nevertheless, computed tomography has better diagnostic value and elucidates the delicate anatomy of the bronchopulmonary tree [7]. However, bronchoscopy is used to provide a definitive view [8].

The management of tracheal bronchus is decided according to the severity of the presenting symptoms. For instance, removal of the accessory bronchus is the major management option for patients in whom the tracheal bronchus is the cause of recurrent respiratory infections [5].

There is only one other case of incidentally discovered tracheal bronchus that was found after inhalation of a foreign body as Alghamdi et al. reported a case of a six-year-old child who was admitted to the emergency department with complaints of severe shortness of breath and difficulty in maintaining his airway. The foreign body was removed and a tracheal bronchus was observed [7].

## Conclusions

The tracheal bronchus is a rare congenital anomaly, with most cases being asymptomatic. Otolaryngologists, anesthesiologists, intensivists, and pediatricians may encounter these cases. Reporting such a rare condition is crucial for diagnosis, management, and avoidance of complications.
